# Simulation-based training for determination of brain death by pediatric healthcare providers

**DOI:** 10.1186/s40064-015-1211-4

**Published:** 2015-08-12

**Authors:** Takashi Araki, Hiroyuki Yokota, Kotaro Ichikawa, Toshio Osamura, Akira Satomi, Tomomitsu Tsuru, Minoru Umehara, Takehiro Niitsu, Tsuyoshi Yamamoto, Kazutaka Nishiyama

**Affiliations:** Department of Emergency and Critical Care Medicine, Nippon Medical School Hospital, Sendagi 1-1-5, Bunkyo-ku, Tokyo, 113-8603 Japan; The Exploratory Committee for Brain Death Determination and Related Issues, The Japanese Society of Emergency Pediatrics, Shinjuku 1-15-11, Shinjuku-ku, Tokyo, 160-0022 Japan

**Keywords:** Brain death, Determination, Pediatric healthcare providers, Training, Simulation

## Abstract

**Background:**

Low competency for determination of brain death (BD) and unfamiliarity with Japanese BD (JBD) criteria among pediatricians were highlighted in previous nationwide studies. Because the JBD criteria were amended in 2010 to allow organ donation from pediatric brain-dead donors, we created a 2-day training course to assess knowledge and improve skill in the determination and diagnosis of pediatric BD.

**Methods:**

The course consisted of two modules: a multistation round session and a group discussion session, and was bookended by a before and after 20-question test. In the multistation round session, participants rotated between stations staffed by expert faculty members. For hands-on skill development, we used the Sim Junior 3G™ simulation mannequin (Laerdal Medical, Wappingers Falls, NY, USA) for structured simulations. In the group discussion session, we implemented simulation-based role playing to practice decision making in prepared scenarios of complicated clinical situations. We investigated the participants’ impressions of the course by self-scoring and questionnaires.

**Results:**

Of 147 pediatric healthcare providers from multiple specialties who participated in this course, 145 completed the entire process. The course was evaluated in three aspects with self-scoring and questionnaires: (1) value (4.58 ± 0.64; range 1–5); (2) time schedule (2.40 ± 0.61; range 1–3); and (3) difficulty (2.89 ± 0.43; range 1–5). Finally, participants scored the entire course program (9.64 ± 1.69; range 1–11). Various positive feedbacks were obtained from a total of 93 participants. Post-test scores (83.6 %) were significantly higher than pre-test scores (62.9 %).

**Conclusion:**

This simulation-based course represents an effective method to train pediatric healthcare providers in determining BD in Japan and may improve baseline knowledge of BD among participants.

## Background

Japan legalized brain death (BD) as human death only when the patient was to be an organ donor. This created a double standard (Aita 2011) of human death after decades of nationwide debate. According to the Japanese BD (JBD) criteria, organ donation must be offered to the family after the patient is clinically determined to be in “a state that can be considered to be BD” (Considered BD, or CBD). The state of CBD is clinically determined when the patients fulfill all of the Japanese BD (JBD) criteria in the absence of apnea testing. If the patient’s consent to be an organ donor was approved under those circumstances, his or her legal death is finally determined with completion of Legal BD (LBD) examinations at two different times (Natori 2011).

The JBD criteria were amended in 2010 to also allow children younger than 16 years to become potential candidates for organ donation. One of the most significant changes in the JBD criteria prohibits organ donation from children having a history of any type of child abuse or abusive head trauma as the primary cause of brain damage; this is because abusive parents cannot act as a child’s legal representative (Yamada et al. 2010).

Despite revision of the JBD criteria, only seven children younger than 16 years were diagnosed with LBD for organ donation over the past 4 years. Several explanations are possible for this phenomenon. The Japanese Association of Neurological Surgeons investigated the current preparedness for juvenile LBD examination in both academic and non-academic neurosurgical institutes through mail-based questionnaires, the results of which revealed that only 17 % of the institutes had a specific diagnostic team for BD (Nagahiro and Teramoto 2010). Also, only 154 designated pediatric intensive care units (PICU) beds are available for the entire Japanese pediatric population (Nakawaga 2010). Furthermore, physicians, especially those involved in pediatric care, have not been evaluated for clinical competence to perform BD examinations.

In 2011, we created a 2-day training course to provide knowledge and practical information on BD examination and related issues. This course contains structured simulation stations, lectures, and case studies presented by expert faculty members. We chose a simulation-based role play discussion to improve specific clinical decision making for organ donation after determining BD. This structure also allowed us to evaluate the participants’ familiarity with the BD determination and their understanding of related issues by self-scoring tests at the end of the course.

## Methods

The course was designed to train pediatric healthcare providers in determining BD based on the JBD criteria practice parameters in two separate modules: a multistation round session and a group discussion session.

### Baseline knowledge assessment

All participants took 20-question pre- and post-tests. The pre-test was taken before the keynote lectures to assess baseline knowledge, and the post-test was performed after the group discussion to assess improvement in knowledge. All questions were related to the entire program content.

### Keynote lecture

Two lectures were provided for improving baseline knowledge; (1) the current situation of organ donation from BD donors in Japan; and (2) the history, definition, and pathophysiology of BD.

### Multistation round

This session covered six different topics of BD determination for pediatric patients: (1) clinical examination of LBD following the JBD criteria, (2) apnea testing, (3) ancillary testing using electroencephalogram (EEG), (4) psychological care for family members, (5) the process of organ donation, (6) how to detect abused children. The participants rotated among these stations every 25 min with a predetermined time schedule. For hands-on skill development, we used the Sim Junior 3G™ simulation mannequin (Laerdal Medical, Wappingers Falls, NY, USA). This model is accompanied by a monitor that can display various vital signs such as heart rate, oxygen saturation, blood pressure, temperature, respiratory rate, and end tidal CO_2_.

#### Preparation

We intubated the mannequin with a 6.0-mm endotracheal tube and provided a flashlight for pupillary reflex assessment, cotton swabs for corneal reflex assessment, an 18-G needle as noxious stimuli to the neck for ciliospinal reflex assessment, a 30-cc syringe with a suction catheter for oculovestibular reflex assessment, and a laryngoscope and suction catheter for gag reflex assessment. A suction catheter, an oxygen tube, and a Jackson-Rees circuit were provided for the apnea test. An EEG machine (Nihon Kohden, Tokyo, Japan) was used for a practical presentation about the artifacts of EEG recordings. Staff members prepared the environment in each station and the timekeeper strictly managed the timetable for comfortable rotation. The experts conducted the session from orientation to debriefing.

All participants were given a booklet that included copies of all session slides for self-study, six case scenarios for group discussion, and a self-scoring sheet.

#### Clinical examination

After hearing a brief summary of JBD criteria, the participants were told to perform a complete BD examination while verbalizing their thorough examination process. The apnea test was performed last. The facilitator instructed the participants to track the JBD criteria in the booklet and to self-evaluate their performance.

#### The apnea test

After a short orientation regarding the appropriate apnea testing procedure, the facilitator demonstrated the entire procedure with a child mannequin. The participants were asked to answer brief questions about the pitfalls of apnea testing.

#### EEG recording

In a hands-on session, the participants were asked to place the electrodes on a partner’s arm to learn the effect of recording artifact on the fivefold sensitivity of the EEG. After this demonstration, the participants could recognize that the EEG recording requires a sophisticated technique to obtain a flat line on brain-dead patients.

#### Psychological care for the family

Based on the results of lecturers’ interviews with the family members of brain-dead patients, the participants received a short lecture about the psychological process of the patient’s family to accept the BD determination. The participants were instructed to be aware of the importance of psychological support of medical staff.

#### Role of the Japan Organ Transplant Network (JOTNW)

The participants received detailed information about the role of the JOTNW and the epidemiology of organ transplantation in Japan to understand the correct process for contacting this organization when potential donors are identified.

#### How to determine a case of child abuse

Per the JBD criteria, parents who are suspected to have abused their children cannot act as legal representatives. Therefore, organ procurement from children with a history of child abuse will not be performed even if a clinical determination of BD is made. The clinical approach to child abuse is not always straightforward and requires multidisciplinary investigation. After this session, the participants were expected to have the comprehensive knowledge necessary for diagnosis of child abuse.

### Group discussion

#### Case presentation

A clinical vignette of a child with a history of devastating brain injury was provided to each group for discussion of whether the patient would be an appropriate candidate for LBD determination. The participants were required to consider various related issues and to relay their final decision.

*Example* A 16-year-old girl has a long and complex history of glioblastoma multiforme that has required four tumor resection operations and postoperative chemotherapies for the past 12 months. The patient’s current prognosis is terminal and she has been prescribed full-time bed rest. The patient is able to open her eyes and move her limbs but is not able to communicate verbally. Currently, the patient does not have any evidence of systemic organ failure. The patient experienced a seizure for >5 min and was obtunded. She was subsequently returned to her hometown. The district emergency responders found her in cardiopulmonary arrest status. The patient was resuscitated and transferred to the nearest emergency center, where she was intubated and taken to the intensive care unit for further care. The next day, the patient’s pupils were fixed and dilated, and EEG results showed total electrocerebral silence. The patient had no advance directive, and her family was unable to decide whether to ask you to stop current treatment. Two days later, the patient’s family showed you a memo from her desk at her home. The note read: “I would like to donate all my organs and tissues for patients affected by organ failure when I become brain dead.” It was written a month ago by the patient.

### Self-scoring and questionnaires

**Fig. 1:**
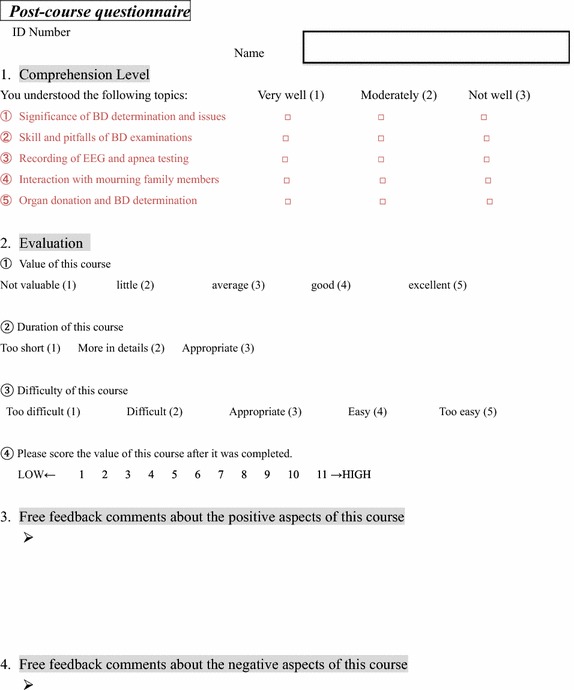
Post course questionnaire.

At the end of the course, all participants answered questionnaires (Fig. 1). The post-course questionnaires comprised three parts: (1) comprehension level, (2) evaluation, and (3) feedback comments. The comprehension level was scored in five aspects on a scale of 1–3: (1) significance of BD determination and related issues, (2) skill in determining BD upon examination, (3) recording of EEG and apnea testing, (4) interaction with mourning family members, and (5) knowledge of organ procurement. For course evaluation, participants were asked to score in three aspects: value and difficulty of the course on a scale of 1–5, duration of the course on a scale of 1–3. Finally, the entire program of this course was scored on a scale of 1–11. In the post-course feedback comments, we received various opinions from participants. The data were presented as mean score ± standard deviation (SD).

### Statistical analysis

Test scores between groups were compared using Student’s t-test for continuous variables. Statistical significance was established at p < 0.05.

## Results

**Fig. 2:**
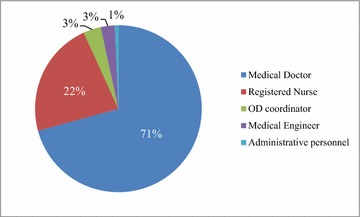
Occupation of participants. *OD* organ donation.

**Fig. 3:**
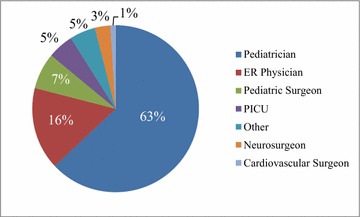
Specialties of Physicians attending the course. Other: anesthesiologist (2), general practitioner (1), medical student (1). *ER* emergency room, *PICU* pediatric intensive care.

A total of 147 participants have taken this course in the past 4 years, and 145 of these have completed the entire program. The highest participation rates came from medical doctors (n = 104; 71 %), followed by registered nurses (n = 33; 22 %), organ donation (OD) coordinators (n = 5; 3 %), medical engineers (n = 5; 3 %) and administrative personnel (n = 1; 1 %) (Fig. 2). Among 104 medical doctors, the highest participation rates came from pediatric specialists (66/63 %), followed by emergency medicine (17/16 %), pediatric surgery (7/7 %), PICU (5/5 %), other (5/5 %), and neurosurgery (3/3 %) specialists (Fig. 3).

**Fig. 4:**
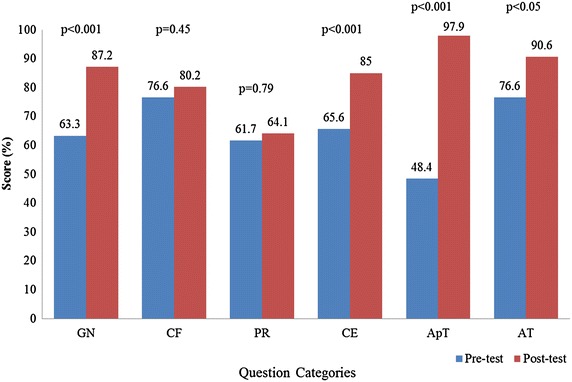
Pre-test and post-test scores by question categories. *GN* general knowledge, *CF* confounders, *PR* prerequisites, *CE* clinical examination, *ApT* apnea testing, *AT* ancillary test.

The post-test scores were significantly higher than the pre-test scores (Fig. 4), increasing from a mean of 62.9 % to a mean of 83.6 % (p < 0.001). In the categories of confounders and prerequisites, there was no significant increase in scores, but participants improved in other categories such as general knowledge, clinical examination, apnea testing, and ancillary test.

**Fig. 5:**
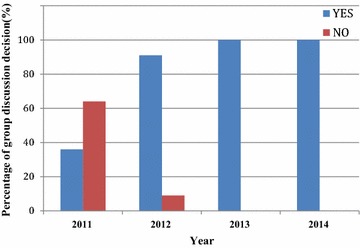
Group discussion decision: should the presented case advance to BD determination for organ donation?

In the “Group discussion”, two of the six groups (33 % of participants) in 2011 decided that the presented case should be considered for BD determination for organ donation. In 2012, four of the six groups (66 % of participants) agreed with organ donation in 2012. All groups in 2013 (one of one, 100 % of participants) and 2014 (six of six; 100 % of participants) determined that the presented case should be considered for BD determination for organ donation (Fig. 5).

Results from the post-course questionnaire revealed the participants’ level of comprehension in three points on a scale of 1–3: (1) significance of BD determination and related issues (2.63 ± 0.48), (2) skill in determining BD upon examination (2.46 ± 0.53), (3) recording of EEG and apnea testing (2.54 ± 0.53), (4) interaction with mourning family members (2.62 ± 0.52), and (5) knowledge of organ procurement (2.62 ± 0.50). The course was also evaluated in three aspects: (1) value (4.58 ± 0.64), (2) time schedule (2.40 ± 0.61), and (3) difficulty (2.89 ± 0.43). Overall, the average score on the 11-point evaluation was very high (9.64 ± 1.69), and the course continued to receive positive evaluations over the 4 years.

In the post-course feedback comments, we received various opinions from participants. As positive feedback, 23 of 93 (25 %) participants stated that they were able to learn the details of neurological examination for BD determination. Second, 20 of 93 (21.5 %) participants mentioned that this course was useful for knowing how to exclude child abuse victims (Table 1).

**Table 1:** Post-course feedback comments

Positive feedback from participants	No. (%)
Able to learn details of neurological examination for BD diagnosis	23 (25)
Learned how to exclude child abuse victims	20 (21.5)
Able to communicate with experts and the other participants	17 (18.3)
Lectures are impressive	17 (18.3)
Able to summarize knowledge about BD criteria	12 (12.9)
Able to recognize the entire process of BD diagnosis	11 (11.8)
Others	3 (3.2)

## Discussion

A main objective of this study was to investigate how often participants were involved in BD determination for severely brain-damaged pediatric patients in general practice. Only ten participants were previously involved in LBD diagnosis for adult organ donors and no participant had experience with BD determination in children. To our knowledge, two previous nationwide studies of pediatric BD in Japan have been conducted and have revealed low competency for determining BD and unfamiliarity with JBD criteria among pediatricians (Takeuchi 2000). In addition, those studies highlighted the need for pediatricians to learn about BD determination in Japan. From our results, 66 of 104 (63 %) medical doctors who participated in this course were pediatric specialists, and all medical doctors from the other departments were originally from pediatric institutes. Moreover, 18 of 33 (55 %) registered nurses were from pediatric departments. Therefore, we assume that this course may be beneficial for pediatric healthcare providers to learn about BD. In his review, Mizuguchi (2010) speculated that Japanese pediatricians believe the following and are unlikely to perform BD determination: (1) strict determination of BD is unnecessary when the patient is younger than 15 years old because the previous law prohibited organ transplant from pediatric BD donors, (2) apnea testing is not required because CBD is sufficient to understand the extent of brain damage and to inform the family of the child’s prognosis, (3) several case reports show that apnea testing can be invasive and can cause deterioration after implementation, and (4) obtaining informed consent for apnea testing from the parents is very difficult because of the possibility of complications. However, since organ donation from pediatric brain-dead donors was legally permitted in 2010, the Japanese Society of Pediatrics (JPS) (Igarashi 2010) has issued a position statement on organ donation from pediatric brain-dead donors. The JPS also agreed with the importance of educating Japanese pediatricians about BD determination. We expect that this course will be recognized as a very useful opportunity for pediatric healthcare providers to learn about BD and related issues in the near future.

Another main objective of this study was to elucidate pediatricians’ opinions about BD diagnosis. The Sim Junior 3G™ simulation mannequin, used for hands-on skill development in the clinical examination and apnea testing sessions, is accompanied by a monitor that can display various vital signs such as heart rate, oxygen saturation, blood pressure, temperature, respiratory rate, and end tidal CO_2_. This mannequin model was quite useful for simulating the patient’s physiological reactions during a BD examination, as evidenced by the high evaluation scores in the clinical examination section and apnea testing. Comments were provided about the benefits of this mannequin, particularly for the clinical examination section. The section entitled *Psychological Care in Brain Death Diagnosis* was highly rated. Many participants commented that this section was quite informative and offered information that is useful to understand the psychological stress of the patients’ family members as well as staff members who had cared for the patients. The contents of this section were meticulously prepared based on the lecturer’s original interview results. The section entitled *Role of the Japan Organ Transplant Network* provided participants with practical knowledge of organ donation and was highly rated as well.

Excluding child abuse victims as candidates for organ donors is a very specific requirement in the JBD criteria. This process does not exist in the criteria for organ donation in any other country. The JBD criteria require the use of a checklist for detecting a suspected child abuse case, but no data exist regarding the accuracy and the sensitivity of determination of child abuse using this procedure, and further investigation is needed. Given the complexity of the examination and the medico-legal ramifications of a child abuse determination, this laxity is a matter of grave significance.

Participants valued this course, felt that the schedule was appropriate, and that the course contents were valuable. Although some negative feedback was received, the post-course questionnaire showed the participants’ comprehension level as high overall.

The aim of this course was to provide a widespread opportunity for simulation of BD determination to pediatric healthcare providers. With continuous effort, we will research and improve our intervention to ensure its effectiveness upon implementation, with the hope of firm social reliability on BD determination and subsequent organ donation from pediatric patients.
